# Atrial Fibrillation Caused by Intractable Hiccups: A Unique Cause and Cure

**DOI:** 10.1155/2022/9381109

**Published:** 2022-01-11

**Authors:** Joshua H. Arnold, Neil Brandon

**Affiliations:** ^1^Department of Medicine, University of Illnois at Chicago, Chicago, IL, USA; ^2^South County Hospital Cardiology, Wakefield, RI, USA

## Abstract

We present the case of a 61-year-old male who developed persistent hiccups concurrently with the onset of atrial fibrillation (AF). The hiccups were refractory to traditional treatment but resolved immediately upon electrical cardioversion (ECV) to normal sinus rhythm (NSR). The patient has remained in NSR and free of hiccups. The potential etiologies for hiccups are numerous and varied, and the management of persistent hiccups can be difficult. Cardiac associations including myocardial infarction and pericarditis have been described, while few cases of first-time onset of atrial fibrillation leading to hiccups have been documented. This case discusses a unique instance demonstrating a connection between hiccups and cardiac pathology and an overview of its management.

## 1. Introduction

Little is known about the incidence and pathophysiology of persistent hiccups, those that last longer than 48 hours. While typically transient, and something experienced by most humans, hiccups may become a disruptor of activities of daily living should they not terminate without intervention. Anecdotal cases of its relation to different pathologies along with a plethora of remedies ranging from physical maneuvers to medications have been reported. However, a direct correlation between new onset atrial fibrillation and the termination of both pathologies by electrical cardioversion have yet to be described.

## 2. Case Presentation

A 61-year-old male with a previous history of the left foot cellulitis and osteomyelitis with methicillin-resistant staphylococcus aureus (MRSA) one year prior to this presentation, arrived to the hospital with redness and swelling of the left great toe. The patient had no significant cardiac history. Prior to arrival to the emergency department, the patient had not felt any pain in the swollen area of the foot, had lived an active lifestyle, and was able to bike 25 miles and walk 3 miles a few days before. He had suffered from chills and a fever to 103 F. Vitals in the emergency department noted a fever of 99.9 F, a systolic blood pressure of 106 mmHg, and a heart rate of 115 bpm. On laboratory tests, his WBC count was 22,600 with 5% band neutrophils and an ESR of 62 mm/hr. He was hypokalemic to 3.2 mmol/L and hyponatremic to 129 mmol/L. Lactic acid was noted to be 3.2 mmol/L and C-reactive protein 333.9 mg/L. The patient was admitted and given piperacillin/tazobactam (Zosyn) as well as vancomycin with the clinical suspicion for sepsis due to osteomyelitis. Blood cultures later grew MRSA. Initial X-ray imaging revealed subcutaneous abscess on the plantar aspect of the left foot as well as evidence suggesting chronic destructive changes of the 1^st^ metatarsal due to either soft tissue cellulitis or osteomyelitis.

**Figure 1:**
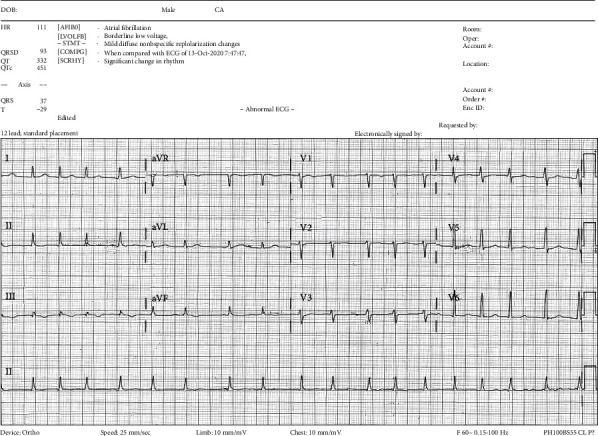
First electrocardiogram revealing atrial fibrillation without any ST-TW abnormalities.

Two days after admission, during the night prior to a scheduled incision and drainage (I&D) of the lesion, the patient developed new onset asymptomatic AF with rapid ventricular response (HR ranging from 110 to 140 bpm) when compared to his presenting EKG that showed sinus tachycardia. He was not aware of his new arrhythmia and denied any feelings of dyspnea, chest pain, or palpitations. No ischemic changes were seen (Figure 1), and diltiazem was prescribed as needed for rate control. The patient has a CHA_2_DS_2_-VASc score of 0, and any decisions on anticoagulation were held pending the operation. Roughly 3 hours after the patient was noted to be in AF, he began to hiccup. The I&D was successful; however, the patient's arrhythmia and hiccups persisted around the clock for five more days. The hiccups were forceful and distressing. The differential diagnosis included central nervous system (CNS) and nerve disorders, metabolic abnormalities, and cardiac or thoracic causes for persistent hiccups.

In hospital, EKG (Figure 1) was noted to show AF without any ST-TW changes. Transthoracic echocardiography revealed low-normal ejection fraction of 58%. Mild-to-moderate mitral regurgitation was seen with mild right atrial enlargement. High-normal pulmonary pressures to 36 mmHg were also detected. No significant structural abnormalities were seen.

**Figure 2:**
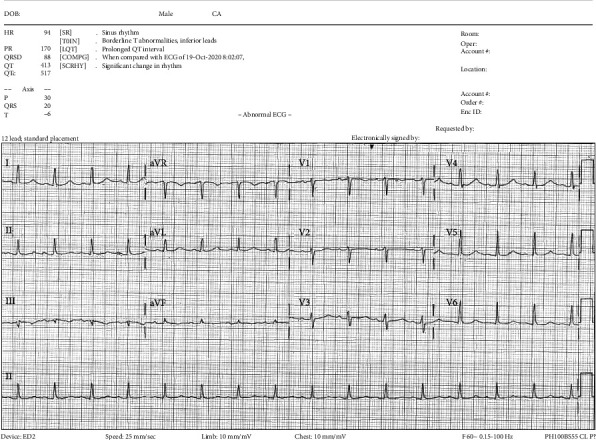
Second electrocardiogram following electrical cardioversion revealing return to normal sinus rhythm.

Physical maneuvers targeted at terminating the persistent hiccups were attempted and unsuccessful. The patient's potassium was repleted without any significant changes noted. Pharmacologic management was then tried including ondansetron, sucralfate, aluminum-magnesium hydroxide (Mylanta), pantoprazole, promethazine, guaifenesin, and several days of baclofen, each of which failed to terminate the hiccups. Five days following the initial onset of AF and hiccups, the patient was scheduled for transesophageal echocardiogram (TEE) and electrical cardioversion (ECV) to restore normal sinus rhythm (NSR). Prior to the procedure, he was noted to have a lot of coughing and hiccupping by the anesthesiologist. The patient was sedated with IV diprivan, and although he continued to hiccup, he maintained a normal oxygen saturation throughout the procedure. TEE showed no evidence of thrombus in the atria or left atrial appendage, and ECV was performed utilizing 200 J of synchronized biphasic energy, administered with a defibrillator via anterior and posterior pads (Medtronic, Minneapolis, MN). Immediately following the electrical charge, the patient returned to NSR (Figure 2) and the patient ceased to hiccup. Upon awaking from sedation, the patient noted marked improvement and complete resolution of hiccups. The patient underwent uncomplicated surgical revision of his total hip prosthesis one month after the AF episode. He did not encounter any perioperative arrhythmias. Four months after his presentation, he has not had any recurrence of AF or of hiccups.

## 3. Discussion

Transient hiccups, although unwanted, tend not to pose any long-term threats to the health of patients. However, prolonged persistent hiccups can decrease quality of life through the interruption of activities of daily living and can portend serious disease. Typically, hiccups are a self-limited phenomenon; however, they can persist. Symptoms lasting longer than 48 hours are considered persistent, while symptoms lasting seconds to minutes or hiccups resolution prior to 48 hours are considered transient. Although the pathophysiology is unclear, it is thought to be related to an involuntary reflex arc of several neural pathways leading to a spasmodic contraction of the diaphragm [1]. Potential etiologies include a long list of central nervous system and nerve disorders, noxious stimuli from metabolic compounds and drugs, and thoracic and postsurgical events. Hiccups related to cardiac anomalies have been explored in several case reports as it relates to myocardial infarction [2], pericarditis [3], and some arrhythmias including bradycardia [4] and AV-block [5, 6], although its direct relation to cardiac arrhythmias has not been well described.

The connection between heart rhythm and hiccups has been documented previously. The phrenic nerve lies adjacent to the left atrium, in close proximity to the entry of the upper pulmonary veins. Likely due to its location, there have been reports of patients coughing during AF ablation procedures. It is possible that an ectopic electrical impulse leading to AF arising in the portion of the atrium near the phrenic nerve may trigger the cough/hiccup reflex in susceptible patients, causing the diaphragm to spasm. It may also be possible that the link between the hiccup reflex arc and heartbeat is a mechanical one, as somatic chest wall mechanoreceptors in the diaphragm or ribcage may respond to an abnormal rhythm [7], thus entering an intractable hiccup cycle.

When symptoms persist, a more thorough medical evaluation is warranted. First-line therapy includes multiple physical maneuvers such as breath holding, valsalva, and pinching one's nose, each of which are designed to break the reflex arc [8]. Certain medications, such as baclofen, metoclopramide, gabapentin, and chlorpromazine, have been reported to break the involuntary spasms in the setting of empiric treatment of persistent hiccups and are considered first-line pharmacotherapy. However, robust clinical data on pharmacotherapy does not exist, preventing the creation of clear guidelines for medical management [9]. Should monotherapy fail, combination therapy is possible, although limited by a patient's ability to tolerate multiple medications based on clinical comorbidities. Other medications such as anticonvulsants, antidepressants, and CNS stimulants have anecdotally been seen to be effective in treatment as well. ECV has been described in one case report to terminate the involuntary contractions of hiccups [10], but the report did not establish a clear association of AF and the onset of hiccups. The authors speculated that the DC CV, rather than the restoration of NSR, had resolved the hiccups. Invasive forms of management include surgical destruction or blocking of the phrenic nerve. Blocking of the C3, C4, and C5 nerve roots has also been shown to permanently terminate hiccups; however, these procedures lead to significant morbidity through paralysis of the hemidiaphragm and are considered extreme.

## 4. Conclusions

In our case report, we describe a patient who, while admitted to the hospital, developed new onset atrial fibrillation (AF) that was followed by persistent hiccups lasting several days that were nonresponsive to traditional therapies, but terminated by ECV. To our knowledge, this is the first report illustrating a direct correlation between new onset AF arrhythmia and persistent hiccups which were terminated by ECV to NSR.
